# Differentiation of multiple myeloma and metastases with apparent diffusion coefficient map histogram analysis

**DOI:** 10.14744/nci.2021.59376

**Published:** 2022-07-05

**Authors:** Murat Baykara, Mustafa Yildirim

**Affiliations:** 1 Department of Radiology, Firat University Faculty of Medicine, Elazig, Turkiye; 2 Department of Radiology, Healthy Science University, Elazig Fethi Sekin City Hospital, Elazig, Turkiye

**Keywords:** Histogram analysis, metastases, multiple myeloma

## Abstract

**OBJECTIVE:**

Multiple myeloma and metastasis are common malignant bone marrow lesions. It may be difficult to distinguish from each other due to similar radiological findings. This study aimed to determine the usefulness of histogram analysis with diffusion-weighted imaging (DWI) in the differentiation of multiple myeloma and metastasis.

**METHODS:**

Twenty patients with multiple myeloma and 20 patients with metastasis who underwent 3T magnetic resonance (MR) imaging with DWI (b=0, 1000 s/mm^2^) were enrolled. All patients had multiple enhancing nodular bone lesions on contrast-enhanced musculoskeletal MR imaging. Histogram analysis was performed from these lesions on the apparent diffusion coefficient (ADC) map. The mean, minimum, median, maximum, standard deviation of the histogram, variance, entropy, uniformity, skewness, kurtosis, size %lower, size %upper, and size %mean values were measured. Results of both groups were compared.

**RESULTS:**

The mean, minimum, median, maximum, standard deviation, and variance values were found to be significantly lower in multiple myeloma than metastasis (p<0.001). When ROC analysis was performed for mean value, the area under the curve=1.000 and when threshold value was selected as 766.076, two groups could be differentiated with 100.0% sensitivity and 100.0% specificity.

**CONCLUSION:**

ADC histogram analysis can be considered as a method to be used in the differentiation of metastases and multiple myeloma.

## Highlight key points

- Multiple myeloma and metastasis are common malignant bone marrow lesions and they have similar radiological findings.
- If the primary tumor history is unknown, radiological differentiation of multiple myeloma and metastasis is difficult.
- ADC histogram analysis showed that the mean, minimum, median, maximum, standard deviation, and variance values were found to be significantly lower in multiple myeloma than metastasis.
- ADC histogram analysis can be used in differentiation of multiple myeloma and metastasis.

Multiple myeloma and metastasis are common malignant diseases involving bone marrow. Multiple myeloma is a malignant tumor of plasma cells that causes lytic bone damage. It is the most common of primary bone tumors and found in the spine, pelvis skull, sternum, and ribs. The radiological appearance of multiple myeloma is irregular lytic lesions of different sizes. Metastatic cancer is the most common malignant secondary bone tumor. Sclerotic, lytic, and mixed type metastases can be seen. Lytic metastases are generally more common. X-ray, computerized tomography, magnetic resonance imaging (MRI) used for diagnosis, and characterization of bone lesions. Metastasis and multiple myeloma have similar radiological imaging findings on MR especially when involving the spine [1, 2].

Diffusion-weighted imaging (DWI) provides information on the degree of cellularity of lesions. Apparent diffusion coefficient (ADC) value provides information on the movement of extracellular water molecules. DWI is widely used for musculoskeletal imaging. DWI and ADC maps may help define the nature of the lesion and provide both qualitative and quantitative information, respectively, on the studied tissue [3].

Microscopic biological changes are associated with tissue heterogeneity. Images used for diagnostic purposes in clinical practice are digital. Each volume element has a value that represents gray-level intensity. Texture analysis is basically a technique that evaluates the signal properties, that is, the gray level intensity and the location of pixels in digital images [4].

Texture properties are mathematical parameters calculated from pixel distribution. It is a post-processing technique that measures gray level intensity, entropy, uniformity, skewness, kurtosis, and pixel distribution histogram. Entropy is one of the tissue analysis parameters and measures the parametric homogeneity in the region of interest (ROI) [5, 6]. It is a parameter that shows inhomogeneity (irregularity) in a histogram. While each data are the same, it is defined as zero. The number increases with the irregular distribution. Uniformity shows how close the image’s gray tones are to the uniform distribution. The number increases with a more uniform distribution [6, 7]. Skewness reflects the asymmetry of the distribution. When the right and left of the center point look the same, the distribution is symmetrical. If the left of the middle has more points, it is positive and vice versa [6]. Kurtosis is a measure of the peak of the distribution. While the histogram is the bell curve, its value is three. If the histogram has a sharper peak, it is >3 [7]. Recently, distribution characteristics of tissue have attracted much attention as diagnostic and a prognostic biomarker [8, 9]. This method has been used in the differentiation of the pathological lesions in the thyroid, liver, kidneys, chest, prostate, brain, lungs, and heart [4, 10].

The aim of our study was to retrospectively determine the usefulness of ADC histogram analysis to differentiate metastasis and multiple myeloma.

## MATERIALS AND METHODS

### Patient Population

**Figure 1:**
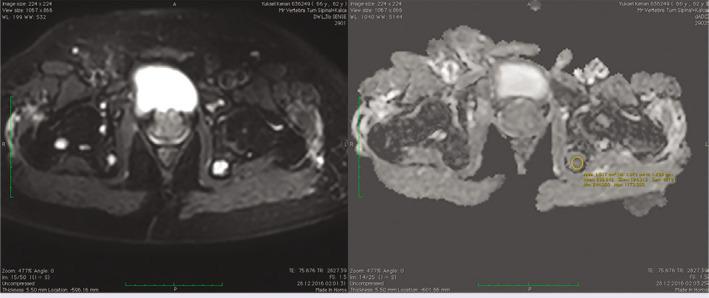
Hyperintense multiple bone lesions on diffusion-weighted imaging in a patient with multiple myeloma and apparent diffusion coefficient histogram analysis.

**Figure 2:**
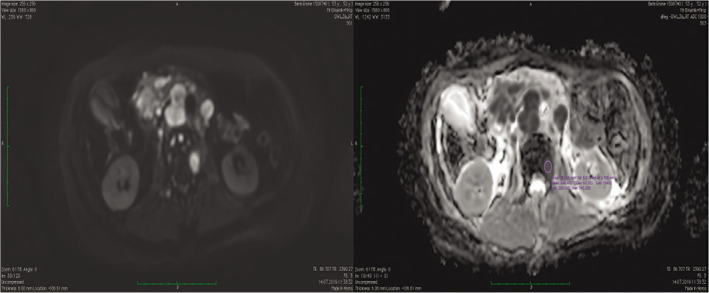
Hyperintense multiple metastatic vertebrae lesions on diffusion-weighted imaging in a patient with lung cancer and apparent diffusion coefficient histogram analysis.

This retrospective study was conducted at the Firat University Faculty of Medicine, Department of Radiology. The study was carried out according to the Declaration of Helsinki Principles. The protocol of the study was approved by the Firat University Non-Interventional Research Ethics Committee (Protocol 2020/15-04). Between 2017 and 2019, previously diagnosed 20 patients with multiple myeloma and 20 patients with primary tumor were included in the study. Multiple bone lesions of patients with primary tumors were accepted as metastasis. Multiple bone lesions of patients with multiple myeloma were accepted as multiple myeloma involvement. All patients had multiple enhancing nodular lesions on contrast-enhanced musculoskeletal MRI. The primary cancers were as follows; lung cancer (n=5), prostate (n=8), and breast cancer (n=7). Histogram analysis was performed on forty lesions (20 for metastasis and 20 for multiple myeloma). A total of 40 DWI and ADC imaging studies for 40 patients were analyzed: Lumbar spine (n=25), thoracic spine (n=14), and pelvic bone (n=1) (Fig. 1, 2).

### MR Imaging Protocols

A 3T MR scanner (Philips Ingenia, Best, Netherlands) was used for imaging with DWI (b=0, 1000 s/mm^2^) sequences. Images were uploaded to a 27-inch iMac computer (Apple Inc., Cupertino, CA, USA). OsiriX V.4.9 imaging software (Pixmeo, Switzerland) was used to measure histogram analysis using the ROI. ROI was drawn to cover most of the lesion in the sections on ADC. Gray level intensity from ROI values, standard deviation of histogram, entropy, uniformity, skewness, kurtosis, size %lower, size %upper, and size %mean (%L, %U, and %M) values were calculated. The entire image analysis algorithm was provided using an in-house program written in MATLAB (version R2009b; MathWorks, Natick, MA, USA).

### Statistical Analysis

Mean±standard deviation was used in expressing the data for statistical analysis was used IBM SPSS for Windows (IBM statistics for Windows version 25, IBM Corporation, Armonk, New York, United States). Chi-square test was used for gender and the Mann–Whitney U test was used to compare the other parameters. Receiver operating characteristic (ROC) analysis was performed for the mean value.

## RESULTS

**Figure 3:**
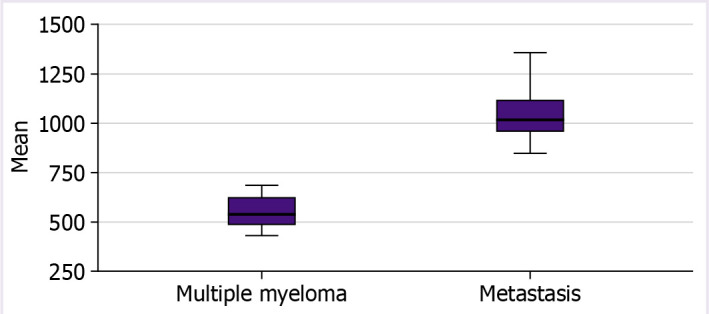
Mean gray level value distributions of groups.

There were no differences between groups in age, gender, entropy, size %L, size %U, size %M, kurtosis, skewness, and uniformity. Mean (Fig. 3), median, minimum, maximum, standard deviation, and variance values were found to be significantly lower in multiple myeloma lesions (p<0.001) (Table 1).

**Table 1:** Quantitative and statistical ADC values of Histogram analysis

	Multiple myeloma (20)		Metastasis (20)		p
	Mean	Stdard deviation	Mean	Stdard deviation	
Age	57.30	12.11	64.10	13.23	0.114
Mean	552.71	74.87	1079.77	223.06	<0.001
Standard deviation	124.99	51.75	211.60	92.89	<0.001
Minimum	278.20	173.50	630.50	297.70	<0.001
Maximum	807.30	141.78	1485.10	250.50	<0.001
Median	556.35	66.99	1083.65	226.90	<0.001
Variance	18166.09	14789.79	52968.94	55493.18	<0.001
Entropy	4.92	0.52	4.90	0.42	0.429
Size %L	14.84	3.26	14.41	4.31	0.738
Size %U	15.89	4.68	14.91	3.37	0.301
Size %M	69.27	5.40	70.68	6.94	0.659
Kurtosis	3.09	0.92	3.07	0.82	0.989
Skewness	-0.11	0.65	0.01	0.63	0.620
Uniformity	0.35	0.12	0.33	0.09	0.779

ADC: Apparent diffusion coefficient.

When ROC analysis was performed for mean value, the area under the curve=1.000, and when threshold value was selected as 766.076, two groups could be differentiated with 100.0% sensitivity and 100.0% specificity.

## DISCUSSION

In older ages, the cause of lytic bone lesions is usually multiple myeloma or metastasis. Both have similar radiological findings [1, 2]. When lytic bone lesions are seen, the primary tumor is investigated if the primary focus is unknown. This study was performed to investigate the value of ADC histogram analysis in the differentiation of multiple myeloma and metastasis.

Myeloma infiltration of the bone marrow is characterized by the highest signal intensities on b-value images compared to normal bone marrow. In addition, bone marrow metastases appear on DWI as high signal intensity areas in an otherwise hypointense vertebral soma. These areas correspond to low signal intensities on ADC maps. Metastasis and multiple myeloma have similar radiological imaging findings on MR especially when involving the spine. In this study, minimum, median, mean, maximum, standard deviation, and variance values were found to be significantly lower in multiple myeloma (p<0.001).

This condition may be related to the histopathological features of multiple myeloma. Multiple myeloma is one of the malignant small round blue cell lesions. Malignant small round cell tumors are characterized by round, small cells, and a high nuclear-cytoplasmic ratio [11]. This arrangement and structure of myeloma cells cause to limit the free water movement in intracellular and extracellular space. Therefore, multiple myeloma lesions showed lower ADC values.

Padhani et al. [12] reported that ADC values of myeloma were lower than breast cancer lesions. In another study, it was reported that the average ADC and minimum ADC value of multiple myeloma were significantly lower than metastatic lesions. In addition, the mean ADC and standard deviation of multiple myeloma were also significantly lower than metastatic lesions [13]. In this study, ADC histogram analysis was performed with the value of b800. Our study was performed with the value of b1000. The sensitivity and specificity of our study were higher. These findings suggest that ADC histogram analysis with a higher b value has higher accuracy in differentiating multiple myeloma from metastases.

Multiple myeloma is a common malignancy in patients above 40 with a male predilection (M:F, 2:1). It arises from red marrow due to the monoclonal proliferation of plasma cells. Bone lesions are mostly encountered in the proximal appendicular skeleton and axial skeleton. Radiologic imaging has an important role in the diagnosis and management of multiple myeloma [14].

Seventy percent of all malignant bone tumors are skeletal metastases. Breast cancer, lung cancer, prostate cancer, and renal cell carcinoma account for approximately 80% of all skeletal metastases [15]. In most cases, the diagnosis of metastatic disease is already known. If no known primary exists, then a bone biopsy can usually allow a definitive diagnosis. However, a bone biopsy is an invasive procedure and has some risks. Therefore, histogram analysis on the ADC map can be performed firstly.

This study has a few limitations. First, the study was performed retrospectively with a small population. Second, metastatic diseases were different from each other. Third, sclerosis and calcifications in the ROI affect ADC.

## Conclusion

As a result, if no known primary focus, ADC and ADC histogram analysis of bone lesions may be useful in the differential diagnosis of metastasis and multiple myeloma. ADC histogram analysis with a higher b value has higher accuracy in differentiating multiple myeloma from metastases.
